# Changes in Admissions to Specialty Addiction Treatment Facilities in California During the COVID-19 Pandemic

**DOI:** 10.1001/jamanetworkopen.2021.17029

**Published:** 2021-07-14

**Authors:** Tami L. Mark, Brent Gibbons, Alan Barnosky, Howard Padwa, Vandana Joshi

**Affiliations:** 1 RTI International, Rockville, Maryland; 2 Department of Integrated Substance Abuse Programs, University of California, Los Angeles

## Abstract

This cohort study examines changes in initiations of treatment in specialty addiction treatment facilities before vs during the COVID-19 pandemic in California.

## Introduction

The COVID-19 pandemic was associated with increased risk of substance use and a surge in fatal drug overdoses.^1,2^ However, little is known about how the pandemic affected addiction treatment utilization.^3,4^ This study evaluated how the initiation of addiction treatment in California changed during the pandemic. We focused on specialty addiction treatment programs because that is where most people receive care for addictions.^5^

## Methods

This cohort study received approval and a waiver of informed consent from the New England IRB and the University of California, Los Angeles, institutional review board. Informed consent was waived because data were deidentified. This study followed the Strengthening the Reporting of Observational Studies in Epidemiology (STROBE) reporting guideline.

This cohort study used data from the 2019 and 2020 California’s Outcomes Measurement System. Alcohol and drug treatment organizations licensed by the California Department of Health Care must collect demographic and medical history information from all patients at treatment initiation using standard data collection forms. California reviews the data to ensure completeness and accuracy. Because of data reporting lags, some California counties had missing data for some months between June and October 31, 2020. The eMethods in the Supplement provide details on missing data and imputation methods.

We identified January 1, 2019, through February 29, 2020, as pre–COVID-19 months and March 1, 2020, to October 31, 2020, as post–COVID-19 months. We estimated linear regressions to calculate the statistical significance of the percentage change in treatment initiations pre– and post–COVID-19 using Stata statistical software version 16.1 (StataCorp). The dependent variable was the ratio of the month’s total initiations to the mean initiations per month over the pre–COVID-19 period. To determine if the percentage change in treatment initiations differed by subpopulations, we added an interaction term between the pre– and post–COVID-19 period and the population characteristic. *P* values were 2-sided, and statistical significance was set at *P* < .05. Data were analyzed from December 2020 through March 2021.

## Results

During the COVID-19 period, monthly initiations were 28.3% (95% CI, −34.9% to −21.7%) lower than they were pre–COVID-19, with mean (SD) monthly initiations of 8994 (713) patients vs 12 544 (920) patients (Figure). The percentage decline in initiations was similar by setting, sex, race, ethnicity, education, whether the patient had children younger than age 18 years, veteran status, and whether the patient was hospitalized before treatment initiation (Table). Larger declines in initiations occurred among individuals without Medicaid coverage (−10.8%; 95% CI, −20.5% to −1.1%), younger than 25 years (compared with those age 25 to 44 years: −15.6%; 95% CI, −25.7% to −5.6%), who were employed (−11.6%; 95% CI, −22.1% to −1.1%), with dependent living (−11.4%; 95% CI, −21.6% to −1.3%), with social supports (−11.8%; 95% CI, −21.4% to −2.2%), without mental illness (−10.5%; 95% CI, −20.0% to −1.1%), with cannabis as the primary drug of abuse (−21.6%; 95% CI, −31.9% to −11.2%), with criminal justice involvement (−13.1%; 95% CI, −22.6% to −3.7%), who had been recently released from prison (−15.9%; 95% CI, −29.6% to −2.2%), who were referred from a driving under the influence program or drug court (–11.5%; 95% CI, –21.9% to –1.1%), or who were referred from another community source (ie, community, child protective services, employer, and self-referral) (−16.1%; 95% CI, −26.5% to −5.7%).

**Figure.**
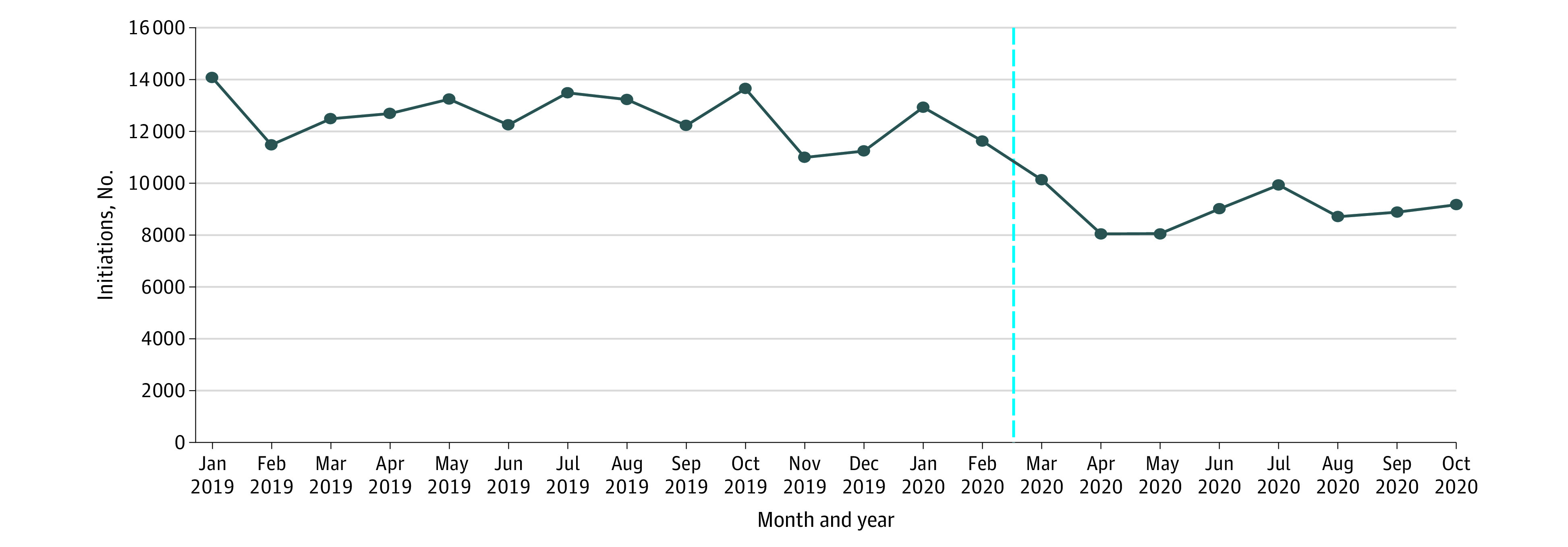
Initiation of Treatment in Specialty Addiction Treatment Facilities in California, January 1, 2019, to October 31, 2020 Dotted blue line indicates the beginning of the COVID-19 period.

**Table.** Mean Monthly Treatment Initiations in Specialty Addiction Treatment Facilities in California, January 1, 2019, to February 29, 2020 (Pre–COVID-19), and March 1, 2020, to October 31, 2020 (Post–COVID-19)

Subpopulation	Initiations per mo, mean (SD), No.		Change, %	Difference (95% CI), %	*P* value
	January 2019-February 2020	March 2019-October 2020			
Type of service					
Outpatient	6025 (483)	4428 (455)	–26.5	0 [Reference]	.57
Residential	3292 (212)	2329 (221)	–29.2	–2.7 (–12.3 to 6.9)	
Sex					
Men	5833 (434)	4163 (383)	–28.6	–3.1 (–12.4 to 6.3)	.51
Women	3484 (243)	2594 (239)	–25.5	0 [Reference]	
Age, y					
<25	1278 (118)	777 (85)	–39.2	–15.6 (–25.7 to –5.6)	.003
25-44	5604 (401)	4284 (349)	–23.6	0 [Reference]	NA
≥45	2435 (188)	1696 (181)	–30.3	–6.8 (–16.8 to 3.2)	.18
Race/ethnicity					
Black, non-Hispanic	871 (81)	621 (65)	–28.7	–2.2 (–12.5 to 8.0)	.66
White, non-Hispanic	4266 (304)	3137 (320)	–26.5	0 [Reference]	NA
Hispanic	3382 (247)	2415 (195)	–28.6	–2.1 (–12.4 to 8.1)	.68
Other^a^	498 (41)	359 (45)	–27.8	–1.4 (–11.6 to 8.9)	.79
Education					
<HS	2695 (209)	1815 (198)	–32.6	–7.8 (–17.3 to 1.6)	.10
HS	4330 (327)	3255 (273)	–24.8	0 [Reference]	NA
>HS	2293 (159)	1687 (150)	–26.4	–1.6 (–11.1 to 7.8)	.73
Parent with children age <18 y					
Yes	3945 (305)	2987 (235)	–24.3	0 [Reference]	.40
No	4971 (368)	3566 (338)	–28.3	–4.0 (–13.5 to 5.5)	
Missing	401 (61)	204 (65)	NA	NA	NA
Veteran					
Yes	228 (28)	159 (22)	–30.5	–3.1 (–15.6 to 9.4)	.62
No	9053 (645)	6574 (577)	–27.4	0 [Reference]	
Missing	36 (14)	25 (10)	NA	NA	NA
Employment status					
Employed	1846 (160)	1200 (193)	–35.0	–11.6 (–22.1 to –1.1)	.03
Unemployed	5549 (360)	4250 (268)	–23.4	0 [Reference]	NA
Not in labor force	1922 (166)	1308 (145)	–31.9	–8.5 (–19.0 to 2.0)	.11
Medicaid					
Yes	7664 (565)	5707 (523)	–25.5	0 [Reference]	.03
No	1650 (136)	1050 (95)	–36.4	–10.8 (–20.5 to –1.1)	
Current living arrangements					
Homeless	2952 (206)	2055 (170)	–30.4	–8.8 (–18.9 to 1.4)	.09
Dependent living	2499 (234)	1673 (155)	–33.1	–11.4 (–21.6 to –1.3)	.03
Independent living	3866 (303)	3030 (331)	–21.6	0 [Reference]	NA
Social support in the past 30 d					
Yes	3841 (269)	2520 (326)	–34.4	–11.8 (–21.4 to –2.2)	.02
No	5476 (417)	4238 (287)	–22.6	0 [Reference]	
Mental illness					
Yes	3754 (258)	2960 (248)	–21.2	0 [Reference]	.03
No	5487 (426)	3749 (372)	–31.7	–10.5 (–20.0 to –1.1)	
Missing	76 (11)	48 (8)	NA	NA	NA
Criminal justice involvement					
Yes	3757 (284)	2430 (260)	–35.3	–13.1 (–22.6 to –3.7)	.008
No	5560 (398)	4327 (357)	–22.2	0 [Reference]	
Prison stay in the past 30 d					
Yes	496 (59)	285 (70)	–42.5	–15.9 (–29.6 to –2.2)	.02
No	8821 (657)	6472 (562)	–26.6	0 [Reference]	
Source of referral					
Health care practitioner	1024 (90)	757 (74)	–26.1	0 [Reference]	NA
School	482 (41)	415 (22)	–14.0	12.1 (1.7 to 22.4)	.02
Probation or parole	4608 (378)	3633 (331)	–21.2	4.9 (–5.5 to 15.3)	.35
DUI/DWI or drug court	2193 (168)	1369 (158)	–37.6	–11.5 (–21.9 to –1.1)	.03
Other community referral	1010 (84)	584 (97)	–42.2	–16.1 (–26.5 to –5.7)	.003
Primary drug					
Alcohol	2164 (162)	1649 (152)	–23.8	0 [Reference]	NA
Opioids	2934 (218)	2293 (254)	–21.8	2.0 (–8.5 to 12.3)	.71
Methamphetamine	2950 (225)	2041 (184)	–30.8	–7.0 (–17.4 to 3.3)	.18
Cannabis	912 (89)	498 (83)	–45.4	–21.6 (–31.9 to –11.2)	<.001
Other	358 (28)	276 (26)	–22.7	1.1 (–9.2 to 11.5)	.83
ED visit in the past 30 d					
Yes	2003 (145)	1394 (147)	–30.4	–3.7 (–13.6 to 6.1)	.45
No	7314 (590)	5364 (471)	–26.7	0 [Reference]	
Hospitalization in the past 30 d					
Yes	992 (91)	636 (89)	–35.9	–9.4 (–20.2 to 1.4)	.09
No	8325 (623)	6121 (522)	–26.5	0 [Reference]	

Abbreviations: ED, Emergency Department; DUI, driving under the influence; DWI, driving while intoxicated; HS, high school; NA, not applicable.

^a^ Includes individuals who identified as Asian or Pacific Islander, American Indian or Alaska Native, and other race.

## Discussion

This cohort study found that the COVID-19 pandemic was associated with a 28% decline in addiction treatment initiations through October 2020. Other research that has examined the association of the pandemic with the use of medical services reported minimal declines in receipt of nonelective procedures and prescriptions but large decreases in receipt of preventive and elective procedures.^6^ Individuals may have been reluctant to seek addiction treatment for fear of becoming infected with SARS-CoV-2. Greater outreach and assurance about the safety of treatment during the pandemic may have allayed these concerns. The decline may also reflect the inability of addiction treatment organizations to treat as many patients as before the pandemic. More rapid and robust government intervention to facilitate the acquisition of personal protective equipment, telehealth equipment, additional staff, and space to deliver socially distanced services may have helped maintain access. Additionally, courts reduced operations during the pandemic, and prisons expedited the release of nonviolent offenders because of the risk of COVID-19 in crowded prison environments. The criminal justice system may need better procedures to connect individuals to addiction treatment during public health emergencies. One limitation of this study was that it only includes specialty addiction services in California; therefore, these findings may not be generalizable and should be replicated using additional states and data from nonspecialty settings. Research is also needed to understand the cause of the decline in initiations and the extent to which it was due to reduced demand for services or reduced ability to supply treatment.

## Notes

Supplement. eMethods.
