# Table Heading Error and Additional Clarifications

**DOI:** 10.1001/jamanetworkopen.2023.18791

**Published:** 2023-05-31

In the Research Letter, “Changes in Admissions to Specialty Addiction Treatment Facilities in California During the COVID-19 Pandemic,”^1^ published in *JAMA Network Open* on July 14, 2021, there was an error in a column heading in the Table. “March 2019” should be “March 2020.” In addition, in response to a reader asking for clarifications, the editors have added the following: the unit of analysis “initiation” has been defined as “admission to either inpatient or outpatient treatment programs;” the number of treatment initiations has been added (“237 835 before imputation and 247 566 after imputation”); and totals have been added to the eTables in the Supplement.^1^ None of the findings, interpretations, or conclusions are affected by this correction and these clarifications.
