# Supplementary material for: Changes in Admissions to Specialty Addiction Treatment Facilities in California During the COVID-19 Pandemic
Source: JAMA Netw Open. 2021 Jul 14;4(7):e2117029. doi: 10.1001/jamanetworkopen.2021.17029 (PMC8280956; doi:10.1001/jamanetworkopen.2021.17029)
Supplement: Supplement. — eMethods. [file jamanetwopen-e2117029-s001.pdf]

Supplemental Online Content

Mark TL, Gibbons B, Barnosky A, Padwa H, Joshi V. Changes in admissions to specialty addiction treatment facilities in California during the COVID-19 pandemic. *JAMA Netw Open*. 2021;4(7):e2117029. doi:10.1001/jamanetworkopen.2021.17029

eMethods.

This supplemental material has been provided by the authors to give readers additional information about their work.

© 2021 Mark TL et al. *JAMA Network Open.*

eMethods.

Table A1 displays the count of total admissions by month, with highlighting for cells that had missing data. We imputed missing data by multiplying the missing county's prior month admissions by the monthly percent change for the sum of admissions in all the non-missing counties. For example, if monthly admissions among reporting counties declined by 10% from May to June, and a missing county had 100 admissions in May, we imputed admissions for June as 90. Excluding Los Angeles, the percent of the imputed admissions was 3% in August, 3% in September, and 5% in October. Including Los Angeles, the percent of imputed values was 19% in June, 19% in July, 22% in August, 22% in September, and 24% in October. **Table A2** displays the count of total admissions by month after imputation. Analysis of differences in the trends in treatment initiation by subpopulation were conducted only using counties that completed data in all months.

© 2021 Mark TL et al. *JAMA Network Open.*

Table A1. Count of total admissions by month

	Jan-19	Feb-19	Mar-19	Apr-19	May-19	Jun-19	Jul-19	Aug-19	Sep-19	Oct-19	Nov-19	Dec-19	Jan-20	Feb-20	Mar-20	Apr-20	May-20	Jun-20	Jul-20	Aug-20	Sep-20	Oct-20
Alameda	620	496	568	536	571	573	601	589	559	645	533	530	592	556	506	381	351	476	450	459	482	462
Alpine	2	0	1	2	1	3	0	1	0	1	0	2	0	4	2	1	4	0	0	0	0	0
Amador	7	9	8	6	6	6	8	10	9	8	4	3	8	5	7	7	2	3	7	7	7	7
Butte	76	74	93	110	99	102	117	100	89	105	61	83	121	84	60	40	59	117	97	97	69	87
Calaveras	22	5	13	19	12	5	10	15	13	12	9	17	20	9	7	7	11	10	8	22	24	14
Colusa	7	5	4	5	6	3	4	4	0	3	2	3	3	1	1	2	3	3	4	8	4	2
ContraCosta	276	276	286	255	282	265	351	312	304	294	257	267	388	252	208	143	177	168	173	162	177	195
DelNorte	7	10	12	9	11	5	8	8	3	10	11	11	11	11	5	6	3	6	7	6	6	9
ElDorado	61	43	52	43	41	33	38	44	27	35	40	30	31	32	13	21	25	36	29	19	21	18
Fresno	1299	428	487	468	486	442	473	461	368	473	356	410	386	311	351	254	236	290	277	258	262	254
Glenn	19	24	18	33	21	22	14	17	11	17	15	8	23	19	19	8	6	27	16	14	12	21
Humboldt	93	83	89	77	96	83	96	59	68	80	66	73	71	68	59	43	49	64	275	132	111	125
Imperial	73	63	84	71	79	59	65	133	121	102	64	52	72	59	51	50	55	57	46	36	59	62
Inyo	1	0	2	1	0	1	1	1	1	0	0	0	0	0	0	0	0	0	0	0	0	0
Kern	319	271	302	315	273	259	313	284	230	292	256	260	305	263	274	201	218	163	196	203	102	0
Kings	57	35	33	48	49	55	49	68	53	45	37	50	41	47	40	13	10	9	12	9	8	11
Lake	24	22	26	33	25	15	60	23	23	23	26	23	21	34	35	4	9	15	11	17	14	16
Lassen	1	1	3	3	2	2	8	6	2	4	2	4	2	4	7	0	0	0	12	0	0	0
LosAngeles	2416	2071	2368	2182	2558	2356	2685	2435	2367	2757	2181	2229	2590	2309	2012	1736	1557	1	1	2	2	3
Madera	31	27	19	16	26	21	28	33	30	33	21	23	32	19	21	28	17	21	20	15	17	12
Marin	157	134	159	145	147	167	153	138	128	144	172	179	174	189	109	96	120	141	125	112	99	94
Mariposa	13	10	7	10	7	8	13	4	11	11	8	7	8	16	7	3	8	3	6	3	0	0
Mendocino	20	15	8	13	14	4	16	3	21	26	8	10	15	12	4	5	0	2	100	29	24	25
Merced	119	85	111	104	86	76	110	96	71	86	82	70	78	73	66	49	55	78	74	75	70	68
Modoc	9	8	10	6	9	13	13	9	10	11	5	5	4	8	6	1	7	9	18	0	0	0
Mono	3	4	3	4	4	7	6	9	4	6	2	3	3	5	5	1	1	2	3	2	2	5
Monterey	118	96	107	98	90	99	103	113	84	110	88	95	113	104	90	49	64	9	78	78	74	105
Napa	63	51	67	83	87	66	76	75	69	86	88	82	78	77	60	32	37	41	47	61	56	75
Nevada	96	82	106	118	113	127	153	135	100	120	109	78	131	94	83	54	82	99	88	89	86	102
Orange	632	555	612	689	677	575	595	661	595	716	612	667	741	713	690	552	521	605	549	638	633	602
Placer	151	116	112	123	127	110	112	118	131	124	106	93	92	98	85	93	65	82	98	88	81	91
Sierra	3	0	0	0	0	0	1	1	0	1	1	0	1	2	3	2	3	6	1	3	2	4
Plumas	12	11	10	3	6	10	9	6	4	15	5	5	4	7	13	12	12	18	2	4	1	1
Riverside	758	588	710	728	758	723	797	804	734	867	673	733	870	743	732	570	637	722	705	696	784	759
Sacramento	547	481	485	525	449	431	430	393	249	201	190	191	208	169	156	226	160	178	110	0	1	3
SanBenito	18	20	16	19	14	19	17	20	24	21	19	13	32	18	21	9	17	12	16	16	8	8
SanBernardino	725	576	601	666	747	606	695	691	640	760	606	591	631	608	468	315	352	344	73	79	109	120
SanDiego	1665	1507	1613	1702	1677	1520	1749	1736	1674	1810	1470	1403	1623	1468	1238	1020	1063	1190	1188	1225	1231	1395
SanFrancisco	498	437	458	394	473	531	489	470	476	482	367	410	419	393	399	287	251	235	261	308	320	356
SanJoaquin	323	255	250	309	376	290	282	302	309	313	200	294	315	266	209	177	205	221	195	214	219	239
SanLuisObispo	129	120	112	137	146	126	99	127	129	140	85	106	138	111	102	108	96	102	113	113	78	89
SanMateo	186	120	40	198	164	182	201	207	182	202	150	30	184	174	129	89	71	99	89	0	0	0
SantaBarbara	227	228	235	301	281	238	242	268	224	248	207	211	221	224	224	170	190	179	202	202	198	194
SantaClara	478	404	462	433	427	418	529	472	467	544	405	441	513	437	315	298	279	325	284	269	247	261
SantaCruz	144	141	145	137	153	181	163	177	142	156	107	103	101	104	81	49	80	96	92	64	119	86
Shasta	139	114	146	152	139	126	137	156	143	152	110	123	135	129	135	114	125	114	594	114	141	120
Siskiyou	24	12	20	17	16	17	16	19	11	15	13	23	22	22	9	8	13	13	38	0	0	0
Solano	124	104	111	132	117	123	125	166	138	129	116	123	128	146	137	85	89	100	452	64	89	86
Sonoma	327	332	390	312	320	327	303	310	315	282	271	257	316	285	212	146	160	154	156	181	201	147
Stanislaus	238	225	205	208	190	184	237	306	256	268	274	264	244	249	204	188	167	201	227	212	228	225
Sutter	0	0	0	0	0	0	0	0	0	0	0	0	0	0	0	0	0	0	0	0	0	0
Tehama	16	6	11	17	20	13	10	17	13	9	11	15	19	18	19	16	9	21	11	7	5	10
Trinity	6	1	3	8	3	3	9	4	3	8	5	7	7	9	5	0	4	7	4	7	7	3
Tulare	264	276	292	301	314	236	293	250	232	260	183	205	248	217	171	89	94	143	120	161	176	194
Tuolumne	16	14	10	5	7	8	9	4	9	12	7	6	11	15	8	5	6	10	6	10	5	5
Ventura	325	315	311	295	334	259	263	254	261	304	217	217	262	228	189	109	156	166	190	143	179	194
Yolo	63	58	39	36	71	76	75	69	61	50	57	81	90	83	56	63	48	52	45	40	54	33
Yuba	31	35	43	29	37	40	28	35	30	25	26	25	33	23	14	6	15	18	13	14	15	12

© 2021 Mark TL et al. *JAMA Network Open.*

Table A2. Count of total admissions by month after imputation

	Jan-19	Feb-19	Mar-19	Apr-19	May-19	Jun-19	Jul-19	Aug-19	Sep-19	Oct-19	Nov-19	Dec-19	Jan-20	Feb-20	Mar-20	Apr-20	May-20	Jun-20	Jul-20	Aug-20	Sep-20	Oct-20
Alameda	620	496	568	536	571	573	601	589	559	645	533	530	592	556	506	381	351	476	450	459	482	462
Alpine	2	0	1	2	1	3	0	1	0	1	0	2	0	4	2	1	4	4	5	4	4	5
Amador	7	9	8	6	6	6	8	10	9	8	4	3	8	5	7	7	2	3	7	7	7	7
Butte	76	74	93	110	99	102	117	100	89	105	61	83	121	84	60	40	59	117	97	97	69	87
Calaveras	22	5	13	19	12	5	10	15	13	12	9	17	20	9	7	7	11	10	8	22	24	14
Colusa	7	5	4	5	6	3	4	4	0	3	2	3	3	1	1	2	3	3	4	8	4	2
ContraCosta	276	276	286	255	282	265	351	312	304	294	257	267	388	252	208	143	177	168	173	162	177	195
DelNorte	7	10	12	9	11	5	8	8	3	10	11	11	11	11	5	6	3	6	7	6	6	9
ElDorado	61	43	52	43	41	33	38	44	27	35	40	30	31	32	13	21	25	36	29	19	21	18
Fresno	1299	428	487	468	486	442	473	461	368	473	356	410	386	311	351	254	236	290	277	258	262	254
Glenn	19	24	18	33	21	22	14	17	11	17	15	8	23	19	19	8	6	27	16	14	12	21
Humboldt	93	83	89	77	96	83	96	59	68	80	66	73	71	68	59	43	49	64	275	132	111	125
Imperial	73	63	84	71	79	59	65	133	121	102	64	52	72	59	51	50	55	57	46	36	59	62
Inyo	1	0	2	1	0	1	1	1	1	0	0	0	0	0	0	0	0	0	0	0	0	0
Kern	319	271	302	315	273	259	313	284	230	292	256	260	305	263	274	201	218	163	196	203	102	105
Kings	57	35	33	48	49	55	49	68	53	45	37	50	41	47	40	13	10	9	12	9	8	11
Lake	24	22	26	33	25	15	60	23	23	23	26	23	21	34	35	4	9	15	11	17	14	16
Lassen	1	1	3	3	2	2	8	6	2	4	2	4	2	4	7	6	6	6	7	6	6	7
LosAngeles	2416	2071	2368	2182	2558	2356	2685	2435	2367	2757	2181	2229	2590	2309	2012	1736	1557	1741	1919	1692	1727	1812
Madera	31	27	19	16	26	21	28	33	30	33	21	23	32	19	21	28	17	21	20	15	17	12
Marin	157	134	159	145	147	167	153	138	128	144	172	179	174	189	109	96	120	141	125	112	99	94
Mariposa	13	10	7	10	7	8	13	4	11	11	8	7	8	16	7	3	8	3	6	3	3	3
Mendocino	20	15	8	13	14	4	16	3	21	26	8	10	15	12	4	5	0	2	100	29	24	25
Merced	119	85	111	104	86	76	110	96	71	86	82	70	78	73	66	49	55	78	74	75	70	68
Modoc	9	8	10	6	9	13	13	9	10	11	5	5	4	8	6	1	7	9	18	16	16	17
Mono	3	4	3	4	4	7	6	9	4	6	2	3	3	5	5	1	1	2	3	2	2	5
Monterey	118	96	107	98	90	99	103	113	84	110	88	95	113	104	90	49	64	9	78	78	74	105
Napa	63	51	67	83	87	66	76	75	69	86	88	82	78	77	60	32	37	41	47	61	56	75
Nevada	96	82	106	118	113	127	153	135	100	120	109	78	131	94	83	54	82	99	88	89	86	102
Orange	632	555	612	689	677	575	595	661	595	716	612	667	741	713	690	552	521	605	549	638	633	602
Placer	151	116	112	123	127	110	112	118	131	124	106	93	92	98	85	93	65	82	98	88	81	91
Sierra	3	0	0	0	0	0	1	1	0	1	1	0	1	2	3	2	3	6	1	3	2	4
Plumas	12	11	10	3	6	10	9	6	4	15	5	5	4	7	13	12	12	18	2	4	1	1
Riverside	758	588	710	728	758	723	797	804	734	867	673	733	870	743	732	570	637	722	705	696	784	759
Sacramento	547	481	485	525	449	431	430	393	249	201	190	191	208	169	156	226	160	178	110	96	98	102
SanBenito	18	20	16	19	14	19	17	20	24	21	19	13	32	18	21	9	17	12	16	16	8	8
SanBernardino	725	576	601	666	747	606	695	691	640	760	606	591	631	608	468	315	352	344	73	79	109	120
SanDiego	1665	1507	1613	1702	1677	1520	1749	1736	1674	1810	1470	1403	1623	1468	1238	1020	1063	1190	1188	1225	1231	1395
SanFrancisco	498	437	458	394	473	531	489	470	476	482	367	410	419	393	399	287	251	235	261	308	320	356
SanJoaquin	323	255	250	309	376	290	282	302	309	313	200	294	315	266	209	177	205	221	195	214	219	239
SanLuisObispo	129	120	112	137	146	126	99	127	129	140	85	106	138	111	102	108	96	102	113	113	78	89
SanMateo	186	120	40	198	164	182	201	207	182	202	150	30	184	174	129	89	71	99	89	78	80	83
SantaBarbara	227	228	235	301	281	238	242	268	224	248	207	211	221	224	224	170	190	179	202	202	198	194
SantaClara	478	404	462	433	427	418	529	472	467	544	405	441	513	437	315	298	279	325	284	269	247	261
SantaCruz	144	141	145	137	153	181	163	177	142	156	107	103	101	104	81	49	80	96	92	64	119	86
Shasta	139	114	146	152	139	126	137	156	143	152	110	123	135	129	135	114	125	114	594	114	141	120
Siskiyou	24	12	20	17	16	17	16	19	11	15	13	23	22	22	9	8	13	13	38	33	34	35
Solano	124	104	111	132	117	123	125	166	138	129	116	123	128	146	137	85	89	100	452	64	89	86
Sonoma	327	332	390	312	320	327	303	310	315	282	271	257	316	285	212	146	160	154	156	181	201	147
Stanislaus	238	225	205	208	190	184	237	306	256	268	274	264	244	249	204	188	167	201	227	212	228	225
Sutter	0	0	0	0	0	0	0	0	0	0	0	0	0	0	0	0	0	0	0	0	0	0
Tehama	16	6	11	17	20	13	10	17	13	9	11	15	19	18	19	16	9	21	11	7	5	10
Trinity	6	1	3	8	3	3	9	4	3	8	5	7	7	9	5	0	4	7	4	7	7	3
Tulare	264	276	292	301	314	236	293	250	232	260	183	205	248	217	171	89	94	143	120	161	176	194
Tuolumne	16	14	10	5	7	8	9	4	9	12	7	6	11	15	8	5	6	10	6	10	5	5
Ventura	325	315	311	295	334	259	263	254	261	304	217	217	262	228	189	109	156	166	190	143	179	194
Yolo	63	58	39	36	71	76	75	69	61	50	57	81	90	83	56	63	48	52	45	40	54	33
Yuba	31	35	43	29	37	40	28	35	30	25	26	25	33	23	14	6	15	18	13	14	15	12

© 2021 Mark TL et al. *JAMA Network Open.*
